# Prognóstico de Insuficiência Cardíaca com Fração de Ejeção Intermediária: Uma História ou uma Versão?

**DOI:** 10.36660/abc.20220170

**Published:** 2022-04-07

**Authors:** Adriana Lopes Latado

**Affiliations:** 1 Universidade Federal da Bahia Faculdade de Medicina da Bahia Salvador BA Brasil Universidade Federal da Bahia – Faculdade de Medicina da Bahia, Salvador, BA – Brasil; 2 Universidade Federal da Bahia Hospital Universitário Professor Edgard Santos Salvador BA Brasil Universidade Federal da Bahia – Hospital Universitário Professor Edgard Santos, Salvador, BA – Brasil

**Keywords:** Doenças Cardiovasculares/fisiopatologia, Insuficiência Cardíaca/fisiopatologia, Insuficiência Cardíaca/epidemiologia, Prognóstico, Volume Sistólico, Fibrilação Atrial, Mortalidade

A categoria “insuficiência cardíaca (IC) com fração de ejeção intermediária” (ICFEi), ou seja, com fração de ejeção do ventrículo esquerdo (FEVE) entre 40-49%, foi descrita pela primeira vez em 2016 nas Diretrizes da Sociedade Europeia de Cardiologia sobre a síndrome.^1^ A partir daí, grande parte da comunidade cardiológica mundial adotou a classificação da IC em três categorias de FEVE (reduzida, intermediária e preservada), incluindo a Sociedade Brasileira de Cardiologia (2018),^2^ apesar das incertezas existentes sobre o real significado da nova classificação e, mais importante, qual seria o impacto da identificação do subgrupo ICFEi na prática clínica. Diferentemente da maioria, a American Heart Association e o American College of Cardiology (2013) têm utilizado a terminologia IC ‘borderline’ com FEVE preservada (ICFEp) para definir pacientes com FEVE entre 41 e 49%, o que não foi atualizado no documento de 2017.^3,4^

Nesse contexto, em 2021, diversas sociedades internacionais de cardiologia publicaram um relatório propondo uma definição e classificação universal da IC. Em relação à classificação por FEVE, embora atrativa do ponto de vista clínico e epidemiológico, os autores revisaram as limitações de seu uso sob diferentes aspectos e propuseram categorias de IC nas quais a estratégia terapêutica seria diferente. ICFEi passou a ser sinônimo de IC com FVE “levemente reduzida”, o que também foi adotado pela atualização das Diretrizes Brasileiras de IC, 2021.^5,6^

Nos últimos anos, um grande volume de pesquisas clínicas tem sido publicado para compreender melhor a população com ICFEi quanto à sua morbidade e prognóstico. Os pacientes classificados como ‘intermediários’ parecem apresentar uma sobreposição nas características clínicas, biomarcadores, achados de imagem cardíaca e desfechos clínicos em comparação com aqueles com IC com FVE reduzida (ICFEr) e ICFEp havendo, entretanto, uma tendência de maior similaridade com pacientes portadores de ICFEr. Pacientes com ICFEi, como ICFEr, são mais jovens do que em ICFEp e apresentam maior prevalência de cardiopatia isquêmica e sexo masculino, enquanto, em geral, apresentam menor proporção de fibrilação atrial.^1,7^ No entanto, essa descrição pode variar dependendo na coorte estudada ou nos cenários clínicos avaliados (por exemplo, pacientes ambulatoriais ou internados).^7^

Em relação aos desfechos clínicos, estudos observaram maior mortalidade total na ICFEr, e os pacientes com ICFEi, em geral, estavam na situação intermediária ou mais próxima dos casos de ICFEp.^8,9^ Por outro lado, uma metanálise recente (2021) de 27 estudos prospectivos encontrou que a mortalidade anual total foi significativamente menor na ICFEi (37,5%) do que na ICFEr (43,7%) e na ICFEp (47,3%). A mortalidade cardiovascular, por sua vez, foi menor na ICFEp, maior na ICFEr e intermediária na ICFEi, grupo que teve a menor incidência de internação por IC.^10^

O prognóstico da IC, por outro lado, não está necessariamente relacionado à FEVE.^5^ A ICFEi representa, em média, 10-20% dos casos de IC e, em muitos pacientes, a FEVE intermediária representa um estado transitório e dinâmico, no qual se pode estar diante de uma recuperação de ICFEr ou de uma piora em direção à ICFEr.^5,11^ O tema ainda é bastante controverso, sendo necessários novos estudos, envolvendo populações de diferentes regiões geográficas e cenários clínicos variados.

Nesta edição, Dutra et al.,^12^ avaliaram o prognóstico de uma coorte ambispectiva de 519 pacientes com IC descompensada internados na unidade de terapia intensiva de um único centro brasileiro durante um seguimento médio de quase três anos.^12^ Do total da amostra, 27,0%, 25,4% e 47,6% tinham ICFEi, ICFEp e ICFEr, respectivamente. A média de idade foi elevada, sendo os pacientes com ICFEi e ICFEr levemente mais jovens do que aqueles com ICFEp, mas sem significância estatística. Semelhante a outros artigos, sexo masculino foi mais frequente em ICFEi e ICFEr, e a fibrilação atrial foi significativamente mais prevalente na ICFEp. A mortalidade intra-hospitalar foi alta (14,5%), predominantemente por causas não cardiovasculares, assim como a mortalidade a longo prazo (52,3%). Os autores observaram menor mortalidade na ICFEi em relação à ICFEr, o que foi estatisticamente significativo. Além disso, finalmente, eles identificaram ‘padrões’ (grupos de variáveis) associados à pior sobrevida, sendo a combinação idade de admissão > 77 anos e necessidade de terapia vasopressora a de pior prognóstico. Demência, IC prévia, readmissão hospitalar e creatinina sérica basal >1,48 mg/dL também foram associados, isoladamente ou em grupos, a maior mortalidade no seguimento tardio.

O estudo de Dutra et al.,^12^ é útil e pertinente para investigar um conteúdo tão atual e controverso em representantes da população brasileira. Algumas limitações impedem conclusões definitivas, a maioria já discutida pelos autores na publicação, mas o estudo acrescenta informações que agregam dados anteriores, também exploratórios em sua maioria, no avanço da compreensão da ICFEi. Em 2021, Petersen et al. publicaram os resultados do seguimento de uma coorte prospectiva (n=380) de IC descompensada internada em hospital terciário do Rio Grande do Sul, Brasil, na qual 31,8%, 16,6% e 51,6% apresentavam ICFEr, ICFEi e ICFEP, respectivamente.^13^ Os pacientes eram mais jovens e apresentaram menor mortalidade hospitalar (7,6%) do que no estudo de Dutra et al.^12^ Para a mortalidade total a longo prazo (desfecho primário), os índices também foram elevados, sem detectar diferenças entre as categorias de IC. A causa cardiovascular foi a principal responsável pelos óbitos observados e, em modelos exploratórios multivariados, a ICFEi e a ICFEr foram associadas a maior risco de mortalidade cardiovascular.

Embora o estudo de Dutra et al.,^12^ não conclua definitivamente sobre as características clínicas, etiológicas ou prognósticas da ICFEi, seus dados alimentam a lacuna de conhecimento sobre esse subgrupo de pacientes com IC. Em breve, esperamos que novas e consistentes evidências científicas nos informem se os pacientes com ICFEi são intermediários de dois extremos ou, de fato, de um subgrupo específico.
