# Supplementary material for: Prognóstico de Insuficiência Cardíaca com Fração de Ejeção Intermediária: Uma História ou uma Versão?
Source: Arq Bras Cardiol. 2022 Apr 7;118(4):701–2. [Article in Portuguese] doi: 10.36660/abc.20220170 (PMC9007011; doi:10.36660/abc.20220170)
Supplement: Translation

# Translation: Prognosis of Heart Failure with Mid-Range Ejection Fraction: A Story or a Version?

**Authors:** Adriana Lopes Latado

**Affiliations:** 1 Brazil Universidade Federal da Bahia - Faculdade de Medicina da Bahia, Salvador, BA – Brazil; 2 Brazil Universidade Federal da Bahia - Hospital Universitário Professor Edgard Santos, Salvador, BA – Brazil

**Keywords:** Cardiovascular Diseases/physiopathology, Heart failure/physiopathology, Prognosis, Heart Failure/epidemiology, Stroke, Atrial Fibrillation, Mortality

The category “heart failure (HF) with mid-range ejection fraction” (HFmrEF), i.e., with left ventricular ejection fraction (LVEF) between 40-49%, was first described in 2016 in the European Society of Cardiology Guidelines on the syndrome.^1^ After that, much of the worldwide cardiology community has adopted HF’s classification into three categories of LVEF (reduced, mid-range and preserved), including the Brazilian Society of Cardiology (2018),^2^ despite existing uncertainties about the real meaning of the new classification and, more importantly, what the identification of HFmrEF subgroup would impact on clinical practice. Unlike most, the American Heart Association and the American College of Cardiology (2013) has used ‘borderline’ HF with preserved LVEF (HFpEF) to define patients with LVEF between 41 and 49%, which was not updated in the document of 2017.^3,4^

In this context, in 2021, different international cardiological societies published a report proposing a universal definition and classification of HF. Regarding the classification by LVEF, although attractive from a clinical and epidemiological point of view, authors reviewed the limitations of its use from different aspects and proposed HF categories in which therapeutic strategy would be different. HFmrEF became synonymous with “lightly reduced” LVEF HF, now including LVEF between 41-49%, which was also adopted by Brazilian HF Guidelines update, 2021.^5,6^

In recent years, a large volume of clinical research has been published to understand better the HFmrEF population concerning its morbidity and prognosis. Patients classified as ‘intermediate’ seem to exhibit an overlap in clinical features, biomarkers, cardiac imaging findings and clinical outcomes compared to those with reduced LVEF HF (HFrEF) and HFpEF. However, there is a tendency towards greater similarity with patients with HFrEF. Patients with HFmrEF, such as HFrEF, are younger than in HFpEF and exhibit a higher prevalence of ischemic heart disease and male gender, while, in general, they have a lower proportion of atrial fibrillation.^1,7^ However, this description may vary depending on the cohort studied or the clinical scenarios evaluated (e.g., outpatients or inpatients).^7^

Regarding clinical outcomes, studies have observed higher total mortality in HFrEF, and patients with HFmrEF, in general, were in the intermediate situation or closer to the cases of HFpEF.^8,9^ On the other hand, a recent meta-analysis (2021) of 27 prospective studies found that total annual mortality was significantly lower in HFmrEF (37.5%) than in HFrEF (43.7%) and HFpEF (47.3%). Cardiovascular mortality, in turn, was lower in HFpEF, higher in HFrEF and intermediate in HFmrEF, the group that had the lowest incidence of hospitalization for HF.^10^

HF prognosis, on the other hand, is not necessarily related to the LVEF.^5^ HFmrEF accounts, on average, for 10-20% of HF cases, and in many patients, intermediate LVEF represents a transitional and dynamic state, in which one can be facing recovery from trough HFrEF or a worsening towards trough HFrEF.^6,11^ The topic is still quite controversial, making new studies necessary, involving populations from different geographic regions and varied clinical scenarios.

In this issue, Dutra et al.^12^ evaluated the prognosis of an ambispective cohort of 519 patients with decompensated HF admitted to the intensive care unit of a single Brazilian center during a mean follow-up of almost three years.^12^ Of the total sample, 27.0%, 25.4% and 47.6% had HFmrEF, HFpEF and HFrEF, respectively. The mean age was high, with patients with HFmrEF and HFrEF slightly younger than those with HFpEF. Like other articles, male gender was more frequent in HFmrEF and HFrEF, and atrial fibrillation was significantly more prevalent in HFpEF. In-hospital mortality was high (14.5%), predominantly for noncardiovascular causes, as was mortality at long-term (52.3%). The authors observed lower mortality in HFmrEF compared to HFrEF, which was statistically significant. Furthermore, finally, they identified ‘patterns’ (groups of variables) associated with worse survival, with the combination of age at admission > 77 years and the need for vasopressor therapy being the one with the worst prognosis. Dementia, prior HF, hospital readmission and baseline serum creatinine >1.48 mg/dL were also associated, alone or in groups, with higher mortality at late follow-up.

The study by Dutra et al.^12^ is useful and pertinent to investigating such a current and controversial content in representatives of the Brazilian population. Some limitations preclude definitive conclusions, most of which have already been discussed by the authors in the publication, but the study adds information that joins previous data, also exploratory for the most part, in advancing the understanding of HFmrEF. In 2021, Petersen et al. published the follow-up results of a prospective cohort (n=380) of decompensated HF admitted to a tertiary hospital in Rio Grande do Sul, Brazil, in which 31.8%, 16.6% and 51.6% had HFrEF, HFmrEF and HFpEF, respectively.^13^ Patients were younger and had lower in-hospital mortality (7.6%) than in the study of Dutra et al.^12^ For total long-term mortality (primary outcome), the rates were also high, without detecting differences between the categories of HF. The cardiovascular cause was the main responsible for these observed deaths, and in exploratory multivariate models, HFmrEF and HFrEF were associated with a higher risk of cardiovascular mortality.

Although the study by Dutra et al.^12^ does not definitively conclude on the clinical, etiological or prognostic characteristics of HFmrEF, their data feed the knowledge gap about this subgroup of patients with HF. Soon, we hope that new and consistent scientific evidence will inform us whether HFmrEF patients are intermediaries of two extremes or, indeed, a specific subgroup.
